# Squamous Cell Carcinoma of the Pancreas: A Case Report

**DOI:** 10.7759/cureus.70247

**Published:** 2024-09-26

**Authors:** Alessandra Nagar, Anitha Rao, Petr Protiva

**Affiliations:** 1 Department of Undergraduate Studies, Northeastern University, Boston, USA; 2 Pathology, William W. Backus Hospital, Norwich, USA; 3 Medicine/Digestive Diseases, Yale University and VA Connecticut Healthcare System, West Haven, USA

**Keywords:** squamous cell carcinoma, tumor imaging, carcinoma pancreas, survival analysis, pancreatic malignancy

## Abstract

Primary squamous cell carcinoma (PSCC) of the pancreas is a rare malignancy with poor prognosis and unclear management. Here, we present the case of a patient with PSCC, review the literature, identify areas of therapeutic uncertainty, and conclude that more clinical and molecular data are needed to improve the prognosis of this rare malignancy.

## Introduction

Primary squamous cell carcinoma (PSCC) of the pancreas is an uncommon malignant histological subtype of pancreatic cancer, accounting for only 0.5-2% of all pancreatic cancers [1,2], with an estimated global annual disease burden of 3,000 cases. This rare cancer originates in the pancreatic ducts [1] and is characterized by malignant squamous cells not typically present in the pancreas [3]. Over 95% of pancreatic cancers arise from exocrine portions of the gland (ductal and acinar cells) and are defined as adenocarcinomas, with treatment options being relatively well established [4]. On the other hand, less is known about the epidemiology, prognosis, outcomes, and management of PSCC. The literature suggests that the incidence of PSCC is increasing and is associated with a poorer prognosis than adenocarcinoma [5,6]. Although uncertain, risk factors associated with the development of PSCC include obesity, smoking, familial history of pancreatic cancer, and chronic pancreatitis [7]. No comprehensive clinical trials have been performed to guide clinicians in the management of this rare disease, creating a need for more clinical data [8]. We present the case of a patient with SCC of the pancreas, describe the appropriate diagnostic evaluation to exclude alternate primary lesions, and provide therapeutic options while reviewing the current literature.

## Case presentation

A 70-year-old Black male presented to the VA Connecticut Healthcare Center with abdominal pain, weight loss, and jaundice over one month. The pain was constant, mid-abdominal radiating to the back, moderate in intensity, and increased with meals, associated with progressive anorexia and weight loss of 10 pounds. His jaundice was accompanied by light-colored stools and fatigue, but he denied fever, chills, rigors, and pruritus.

The patient had a past medical history of diabetes, chronic kidney disease, hypertension, and depression. Physical examination was notable for jaundice, temporal wasting, no signs of cirrhosis, palpable non-tender gallbladder, and mild diffuse upper abdominal tenderness without signs of an acute abdomen. No clinical ascites was noted.

Laboratory investigation revealed new normocytic anemia and cholestasis. Direct bilirubin was 4.7 mg/dL (normal: <1.2 mg/dL), aspartate transaminase was 438 mg/dL (normal: <30 mg/dL), alanine transaminase was 430 mg/dL (normal: <40 mg/dL), alkaline phosphatase was 932 mg/dL (normal: <120 mg/dL), albumin was 2.4 mg/dL (normal: 4-7 mg/dL), and international normalized ratio was 1.1 (normal). The serum cancer antigen 19-9 level was 14, within the normal range (<34).

**Figure 1:**
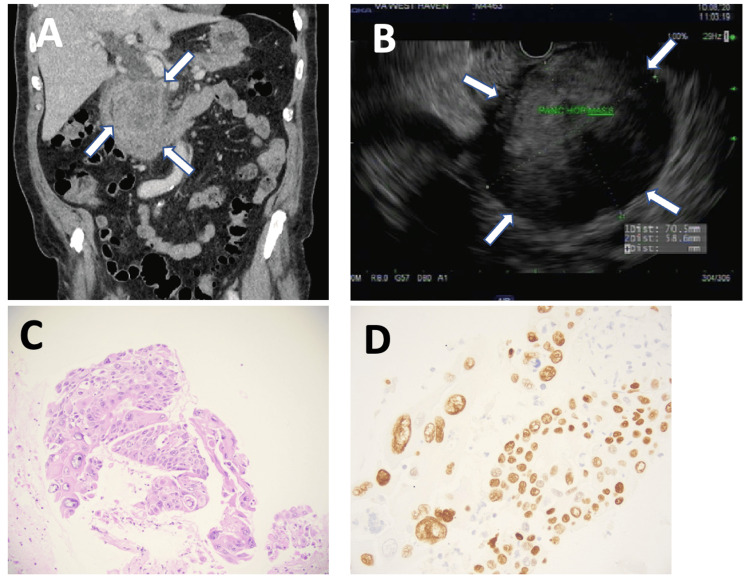
A case of primary squamous carcinoma of the pancreas. A: CT scan of the abdomen demonstrating a 7 cm pancreatic mass causing duodenal and biliary compression (arrows). B: The same neoplasm imaged by endoscopic ultrasound showing a hypodense mass (arrows). C: Hematoxylin-eosin stains of fine-needle biopsy of the mass showed a poorly differentiated tumor with immunostaining positive for cytokeratin and P40 (D: P40 stain is shown), magnification: ×200.

Contrast-enhanced CT imaging showed a 7 cm mass in the pancreatic head causing biliary and pancreatic duct obstruction (Figure 1A). A presumed diagnosis of pancreatic cancer was made, with biliary obstruction and without ascending cholangitis. The patient then underwent endoscopic ultrasound (EUS) for tissue diagnosis. EUS demonstrated a hypodense pancreatic head mass surrounding the superior mesenteric artery, stage cT4N1M0 (Figure 1B). Fine-needle aspiration revealed poorly differentiated SCC, positive for cytokeratin and P40 (Figures 1C, 1D). Genetic analysis demonstrated stable microsatellite instability, mutation burden: 1/MB, *CDKN2A* *P114H*, *KRAS G12V*, *TP53 R273C*, *CCND2*, *FGF23*, *FGF6*, *KDM5A*, and *KRAS* amplifications. Immunohistochemistry demonstrated some loss of *PMS2* nuclear expression.

The patient then underwent endoscopic retrograde cholangiopancreatography (ERCP) to palliate obstruction jaundice. A bile duct stent was placed with the resolution of jaundice.

He then underwent a workup for other primary sites of SCC, which were excluded by endoscopy and cross-sectional imaging. Based on imaging and the stage of the tumor, the patient was managed with definitive chemotherapy. Treatment was initiated with FOLFIRINOX (leucovorin calcium (folinic acid), fluorouracil, irinotecan hydrochloride, and oxaliplatin). Restaging at three months demonstrated disease progression. Despite a change in chemotherapy to gemcitabine and abraxane, the patient died two months later of superior vena cava tumor obstruction and sepsis.

## Discussion

Previous reports of PSCC have demonstrated the rarity of this neoplasm and suggested that pancreatic PSCC has a poorer prognosis than adenocarcinoma [9,10]. A national cancer database review identified 515 patients with SCC of the pancreas and found the median overall survival to be four months, with no difference for gender or location of tumor in the pancreas [10]. However, as with early pancreas adenocarcinoma, they reported that surgery in stages I-II increases overall survival independent of whether the patient underwent chemotherapy, with other studies also indicating surgery as the optimal treatment strategy [2,10,11,12]. Specifically, a systematic review of pancreatic PSCC in 2017 identified 52 patients with SCC of the pancreas, finding resectability and tumor grade associated to be with better outcomes [8]. Furthermore, post-surgery chemotherapy was not associated with increased OS in stages I-II; however, as most patients present at later stages of the disease (14% stage III and 62% stage IV), there is limited data on earlier stages, underlining the importance of early detection [10].

This is relevant because a population-based study identified 214 patients with PSCC of the pancreas and showed rates had increased by 228% from 2000 to 2012. The study identified proportionately more poorly differentiated histology and stage IV disease in SCC compared to adenocarcinoma of the pancreas [5]. Additionally, a national cancer database study suggested that SCC presents at a more advanced stage than adenocarcinoma and is less resectable, presenting challenges in treatment and overall survival that must be addressed [6].

EUS-FNA plays a central role in the diagnosis, providing tissue for histological diagnosis and genetic sequencing. ERCP with biliary stent placement is necessary to palliate jaundice, enabling a full-dose chemotherapy regimen. Genetic analysis of pancreatic cancers defined a squamous subtype with pathways enriched for *TP53* and *KDM6A* mutations, as well as other genetic abnormalities such as those found in PSCC of the head, neck, and lung [13]. As we did not find most of these specific mutations in our patient, more comprehensive sequencing of PSCC cases is needed.

Squamous cells are not typically present in the pancreas, thus it is important to rule out cancer metastasis as the cause of pancreatic SCC, even though metastatic disease to the pancreas comprises fewer than 2% of all pancreatic neoplasms [14]. We ruled out metastatic pancreatic lesions from the more common SCC of the lung, head, neck, and esophagus by upper endoscopy and cross-sectional imaging studies. Imaging characteristics include enhancement of the tumor with contrast and a tumor blush pattern on angiogram [15]. EUS features suggestive of PSCC included well-defined lesions versus diffuse lesions in adenocarcinoma, as seen in our patient.

There is limited data on outcomes and specific therapy for the management of PSCC of the pancreas. Regimens include first-line therapy with gemcitabine and a platinum-based regimen, second-line fluoropyrimidine, and recently neoadjuvant paclitaxel lipid suspension [16,17].

The biology of SCC is distinct from adenocarcinoma and optimal management is not defined. Additional studies examining the interplay between treatment modalities, genetic profiles, and patient characteristics are needed to improve the management of this rare disease.

## Conclusions

The case presentation demonstrated the lethality of PSCC of the pancreas and the therapeutic uncertainty in dealing with this malignancy. A review of the literature suggests that this rare cancer is associated with a worse survival experience compared to adenocarcinoma, highlighting the management challenges and the importance of accurate biopsy and histological assessment. Currently, there is uncertainty about the optimal treatment options, including the choice of chemotherapy, and a lack of standardized guidelines. Surgery in early T1 and T2 lesions is associated with improved survival outcomes, but it remains a viable option only for a limited subset of patients. More clinical and molecular data are urgently needed to enhance treatment strategies and improve the prognosis of this rare and aggressive malignancy.
